# Trends in Home Health Care Among Traditional Medicare Beneficiaries With or Without Dementia

**DOI:** 10.1001/jamanetworkopen.2025.10933

**Published:** 2025-05-16

**Authors:** Rachel M. Werner, Seiyoun Kim, R. Tamara Konetzka

**Affiliations:** 1 Leonard Davis Institute of Health Economics, University of Pennsylvania, Philadelphia; 2 Division of General Internal Medicine, Perelman School of Medicine, University of Pennsylvania, Philadelphia; 3 Center for Health Equity Research and Promotion, Corporal Michael J. Crescenz Veterans Affairs Medical Center, Philadelphia, Pennsylvania; 4 Associate Editor, *JAMA Network Open*; 5 Department of Public Health Sciences, University of Chicago, Chicago, Illinois

## Abstract

**Importance:**

A growing number of people with dementia are opting to live at home and receive care in the community rather than enter a nursing home. Adequately supporting their care at home can be challenging, and Medicare-funded home health care is one common source of care.

**Objective:**

To examine trends in the use of home-based care among traditional Medicare beneficiaries with dementia compared with those without dementia.

**Design, Setting, and Participants:**

A cross-sectional study of home health care use from January 1, 2010, to June 30, 2022, was conducted among 13 604 086 traditional Medicare beneficiaries aged 68 years or older who were receiving home health care during the study period. Statistical analysis took place from February 2024 to March 2025.

**Main Outcomes and Measures:**

Receipt of Medicare-funded home health care, as home health “spells” (the initial Medicare-certified home health episode and all subsequent recertifications were considered 1 home health spell for analysis).

**Results:**

Among the 13 604 086 traditional Medicare beneficiaries included in the study (mean [SD] age, 79.4 [7.7] years; 60.4% women), 27.7% had a diagnosis of dementia. Compared with beneficiaries using home health care without a diagnosis of dementia, those with a diagnosis of dementia were older (mean [SD] age, 82.2 [7.5] vs 78.4 [7.5] years), and a higher percentage were female (61.9% vs 59.9%) and dually eligible for Medicare and Medicaid (17.0% vs 11.1%). There were 30 549 666 new home health spells during the study. Individuals with dementia more commonly used community-initiated home health care than postacute care (53.8% vs 46.2%). Among individuals with a diagnosis of dementia, the initiation of home health spells increased between 2010 and 2019 by 16.8% (from 35.4 to 40.2 spells per 1000 beneficiaries) for community-initiated care and by 21.4% (from 28.9 to 35.1 spells per 1000 beneficiaries) for postacute care. Between 2020 and 2022, home health care use decreased among individuals with dementia; community-initiated spells decreased from 40.2 to 33.6 spells per 1000 beneficiaries, and postacute spells decreased from 35.1 to 28.5 spells per 1000 beneficiaries. In comparison, home health use among individuals without a diagnosis of dementia was relatively low and gradually decreased over the study period: between 2010 and 2019, home health spells decreased by 20.1% (from 8.9 to 7.1 spells per 1000 beneficiaries) for community-initiated care and by 20.7% (from 12.8 to 10.1 spells per 1000 beneficiaries) for postacute care. Home health spells were longer for people with dementia compared with people without dementia (community-initiated care: median, 47 days [IQR, 27-80 days] to 52 days [IQR, 29-89 days] vs 44 days [IQR, 25-76 days] to 50 days [IQR, 27-91 days]; postacute care: median, 40 days [IQR, 23-59 days] to 43 days [IQR, 25-59 days] vs 32 days [IQR, 19-56 days] to 34 days [IQR, 20-56 days]) and increased after 2020 (community-initiated care: median, 55 days [IQR, 33-111 days] vs 53 days [IQR, 27-98 days]; postacute care: median, 48 days [IQR, 27-59 days] vs 42 days [IQR, 24-58 days]).

**Conclusions:**

This study suggests that home health use is high and has increased among people with dementia. Decreasing rates of home health use since 2020 in this high-need population suggest a need for ongoing monitoring of service use and outcomes for people with dementia.

## Key Points

**Question:**

Has the use of Medicare-funded home health care changed for individuals with a diagnosis of dementia?

**Findings:**

In this cross-sectional study of 13 604 086 traditional Medicare beneficiaries aged 68 years or older, use of home health care by individuals with a diagnosis of dementia increased between 2010 and 2019, by 16.8% for community-initiated care and by 21.4% for postacute care. Between 2020 and 2022, use of home health care decreased.

**Meaning:**

This study suggests that home health care is commonly used among individuals with dementia.

## Teaser

This cross-sectional study examines trends in the use of home-based care among traditional Medicare beneficiaries with dementia compared with those without dementia.

## Introduction

In the US, nearly 7 million people live with Alzheimer disease and other dementias,^1^ a number that is expected to increase as the population ages. Although many people with dementia live in nursing homes or other institutional settings, institutional use is decreasing, and a growing majority of people with dementia are opting to live at home and receive care in the community. Home-based care is typically consistent with most people’s preferences and may be particularly important for those with dementia, as institutional settings and transfers can be stressful and disorienting.

Adequately supporting complex care at home for high-need individuals with dementia is challenging, requiring high rates of caregiver assistance and home-based clinical care.^2^ One common source of home-based care is Medicare-funded home health care. Prior studies in small samples have reported high rates of home care among people with dementia.^3,4^ Whether home care use is increasing over time, as institutional care decreases, is unknown. We sought to update prior estimates of the use of home care among older adults with dementia compared with those without dementia, using data through the middle of 2022. We focus on Medicare-funded home care, which provides home-based care for older adults who need skilled care.

## Methods

### Data and Study Sample

We sought to include all beneficiaries enrolled in traditional Medicare and using home health care that was initiated between January 1, 2010, and June 30, 2022, and included all home health use among those beneficiaries. Within this group, we examined 2 cohorts: those with a diagnosis of dementia and those without a diagnosis of dementia. This study was reviewed and approved by the institutional review board at the University of Pennsylvania, which waived participant consent due to the low-risk nature of this retrospective research based on existing data. We followed the Strengthening the Reporting of Observational Studies in Epidemiology (STROBE) reporting guideline for cross-sectional studies.

We used 3 data sources. First, we identified all home health episodes using the 100% Outcome and Assessment Information Set (OASIS) data. We used OASIS to identify home health use as it includes all home health assessments provided by Medicare-certified home health agencies.

We then linked these data with the Master Beneficiary Summary File (MBSF), which is used to identify beneficiaries’ sociodemographic characteristics, including traditional Medicare enrollment, and, using the chronic conditions segment of the MBSF, a diagnosis of Alzheimer disease and related dementias (ADRD). The indicator of ADRD diagnosis is derived from *International Classification of Diseases, Ninth Revision*; *International Statistical Classification of Diseases and Related Health Problems, Tenth Revision*; *Current Procedural Terminology*; and Healthcare Common Procedure Coding System codes from the prior 3 years on any inpatient, skilled nursing facility, home health, outpatient, or clinician claims with specific diagnosis codes.

Finally, we linked these data to 100% MedPAR (Medicare Provider Analysis and Review) claims to identify all discharges from institutional settings, including acute care hospital, skilled nursing facility, inpatient rehabilitation facility, or another institutional setting (eg, long-term acute care hospital). This enabled us to differentiate home health care that was initiated after discharge from an institutional setting vs from the community.

We included all home health use among Medicare enrollees aged 68 years or older, which allowed a 3-year lookback for a diagnosis of dementia. We limited the sample to home health use for beneficiaries enrolled in traditional Medicare at the time of home health initiation, as the diagnosis of ADRD relies on claims data from traditional Medicare. We defined traditional Medicare enrollment as enrolled in Parts A and B and not enrolled in a Medicare Advantage plan (labeled as “HMO” in the MBSF).

### Measures of Home Health Care Use

Our unit of analysis was the home health “spell.” In sensitivity analysis, our unit of analysis was the beneficiaries using home health care. During most of the study period, Medicare defined a home health episode as lasting 60 days. Episodes changed to 30 days after Medicare implemented the Patient-Driven Groupings Model (PDGM) for payment of home health services, starting on January 1, 2020. Both before and after the implementation of PDGM, home health episodes could be recertified as many times as needed if the beneficiary was still eligible for home health care. For all analyses, we created home health spells in which we included the initial Medicare-certified episode and all subsequent recertifications as 1 home health spell.

We classified each home health spell as either postacute care or community-initiated care. Those that were initiated within 14 days of discharge from an institutional setting (eg, acute care hospital, skilled nursing facility, inpatient rehabilitation facility, or another institutional setting) were classified as postacute care. All other home health spells were classified as community initiated. Although there is no established method for defining postacute vs community-initiated home health, we followed a method used in prior work.^5,6,7^ We also measured the median of the total number of days in each home health spell, using the start of care and discharge dates from OASIS.

### Statistical Analysis

Statistical analysis took place from February 2024 to March 2025. We report trends in the rate of home health spells or in the number of home health spells per 1000 people enrolled in traditional Medicare with a diagnosis of dementia and the number of home health spells per 1000 people enrolled in traditional Medicare without a diagnosis of dementia to account for the decreasing number of people enrolled in traditional Medicare over this period.

In sensitivity analyses, we examined trends in home health care use using the Medicare beneficiary, rather than the home health spell, as the unit of analysis because each beneficiary could have more than 1 home health spell. We therefore examined whether any observed trends could be associated with changes in the number of home health spells per beneficiary over the study period.

In addition, given the changes in the population enrolled in traditional Medicare over this period of growth of Medicare Advantage, in a sensitivity analysis, we adjusted rates of home health care use for patient sociodemographic characteristics. To do so, we first aggregated both the numerator (the number of home health spells) and denominator (the number of individuals enrolled in traditional Medicare) to the county-quarter level. We then used individual-level sociodemographic data from MBSF to calculate quarterly county-level means of age, sex, race and ethnicity, and dual eligibility of enrollees in traditional Medicare. Race and ethnicity (as defined in Centers for Medicare & Medicaid Services data) were self-reported by beneficiaries during Medicare enrollment and included Hispanic, non-Hispanic Black (hereafter, *Black*), non-Hispanic White (hereafter, *White*), and other (including American Indian or Alaska Native and Asian or Pacific Islander) categories. Race and ethnicity were included in this study as they are associated with changes in enrollment in traditional Medicare and Medicare Advantage. We used linear regression to adjust the county-level rates of home health spells for these beneficiary characteristics plus county and time fixed effects, clustering SEs at the county level. The regression analyses were weighted by the county-level number of traditional Medicare enrollees.

## Results

We included 13 604 086 Medicare beneficiaries (mean [SD] age, 79.4 [7.7] years; 60.4% women and 39.6% men; 8.5% Black beneficiaries, 1.9% Hispanic beneficiaries, 85.9% White beneficiaries, and 5.7% beneficiaries of other races and ethnicities) who used home health care between January 1, 2010, and June 30, 2022, of whom 27.7% had a diagnosis of dementia (Table 1). Compared with beneficiaries using home health care without a diagnosis of dementia, those with a diagnosis of dementia were older (mean [SD] age, 82.2 [7.5] vs 78.4 [7.5] years), and a higher percentage were Black (9.5% vs 8.1%), female (61.9% vs 59.9%), and dually eligible for Medicare and Medicaid (17.0% vs 11.1%).

**Table 1.** Description of the Study Cohort Overall and by Whether the Individual Had a Diagnosis of Dementia^a^

Characteristic	All traditional Medicare beneficiaries (N = 13 604 086 [100%])	Medicare beneficiaries with a diagnosis of dementia (n = 3 631 678 [27.7%])	Medicare beneficiaries without a diagnosis of dementia (n = 9 972 408 [73.3%])
Age, mean (SD), y	79.4 (7.7)	82.2 (7.5)	78.4 (7.5)
Race and ethnicity, No. (%)			
Black	1 156 755 (8.5)	346 093 (9.5)	810 662 (8.1)
Hispanic	263 046 (1.9)	90 553 (2.5)	172 493 (1.7)
White	11 679 111 (85.9)	3 059 593 (84.3)	8 619 518 (86.4)
Other	768 220 (5.7)	225 992 (6.2)	542 228 (5.4)
Sex, No. (%)^b^			
Female	8 218 904 (60.4)	2 249 050 (61.9)	5 969 854 (59.9)
Male	5 385 181 (39.6)	1 382 628 (38.1)	4 002 553 (40.1)
Dually eligible, No. (%)	1 724 413 (12.7)	616 265 (17.0)	1 108 148 (11.1)

^a^ Description of the study cohort overall and by whether the individual had a diagnosis of dementia is at the level of the unique beneficiary.

^b^ One observation was recorded as “unknown.”

There were 30 998 653 home health care spells between 2010 and 2022, or a mean (SD) of 2.2 (2.0) home health spells per beneficiary. Home health care was more commonly initiated from the community than for postacute care among individuals with a diagnosis of dementia (53.8% vs 46.2%; Table 2). The opposite pattern was observed for those without a diagnosis of dementia (41.3% of home health spells were initiated in the community vs 58.7% for postacute care).

**Table 2.** Number of Home Health Spells Over the Study Period

Home health care spells	All traditional Medicare beneficiaries (N = 30 998 653)	Medicare beneficiaries with a diagnosis of dementia (n = 12 073 801)	Medicare beneficiaries without a diagnosis of dementia (n = 18 924 852)
Community initiated, No. (%)	14 305 970 (46.2)	6 489 697 (53.8)	7 816 273 (41.3)
Postacute, No. (%)	16 692 683 (53.9)	5 584 104 (46.3)	11 108 579 (58.7)

**Figure 1.**
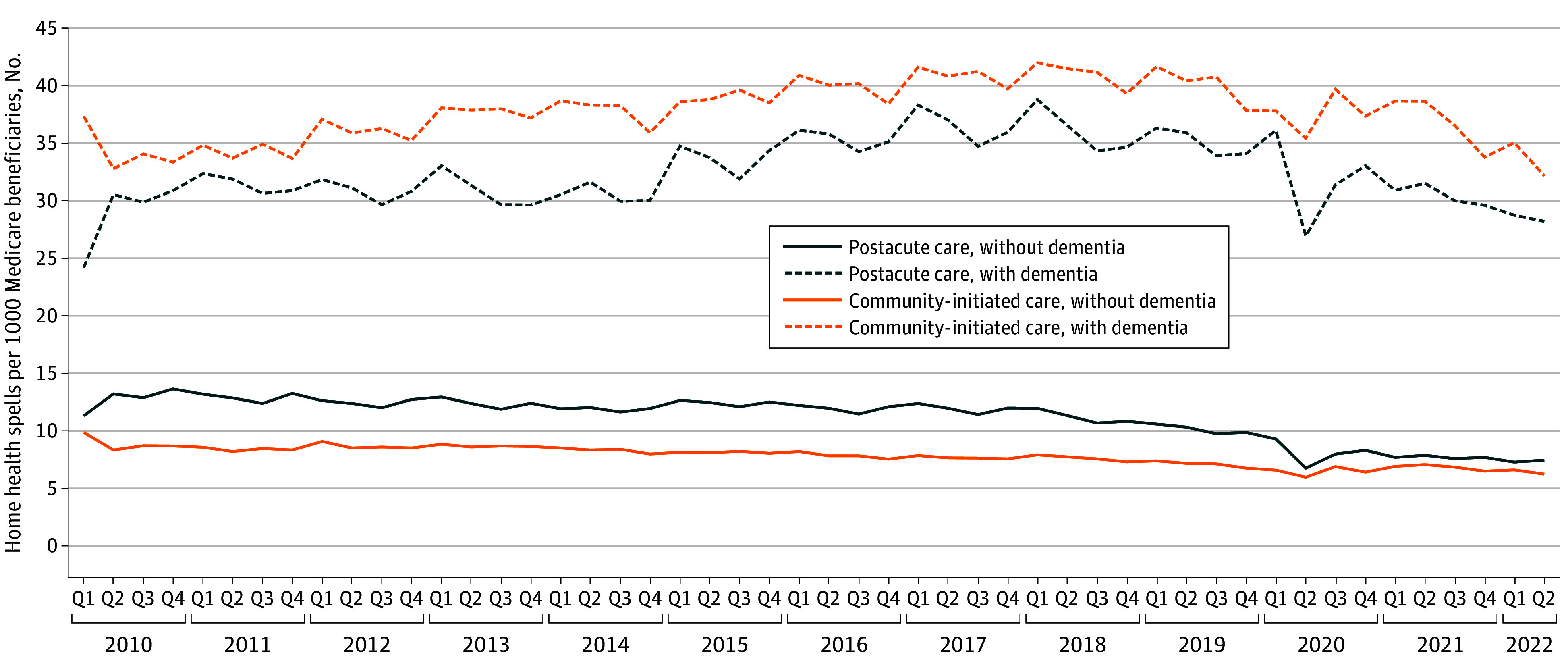
Number of Home Health Spells per 1000 Medicare Beneficiaries With or Without a Diagnosis of Dementia Q indicates quarter.

The initiation of home health spells increased between 2010 and 2019 and then decreased between 2020 and mid-2022 among people with a diagnosis of dementia (Figure 1). Community-initiated spells increased from 35.4 to 40.2 spells per 1000 Medicare beneficiaries with a diagnosis of dementia between 2010 and 2019 (an increase of 16.8%) and then decreased to 33.6 spells in mid-2022. In a similar pattern, the use of postacute home health care increased from 28.9 to 35.1 spells per 1000 beneficiaries with a diagnosis of dementia between 2010 and 2019 (an increase of 21.4%) before decreasing to 28.5 spells in mid-2022.

The level of use and the observed pattern of home health use over time were different for those without a diagnosis of dementia. The initiation of both community-initiated home health care and postacute care was lower for Medicare beneficiaries without a diagnosis of dementia and decreased over this time period: between 2010 and 2019, home health spells decreased by 20.1% (from 8.9 to 7.1 spells per 1000 beneficiaries) for community-initiated care and by 20.7% (from 12.8 to 10.1 spells per 1000 beneficiaries) for postacute care. Trends in the number of beneficiaries using home health care were similar to the observed trends in the number of new home health spells initiated over this time period (eFigure 1 in Supplement 1).

**Figure 2.**
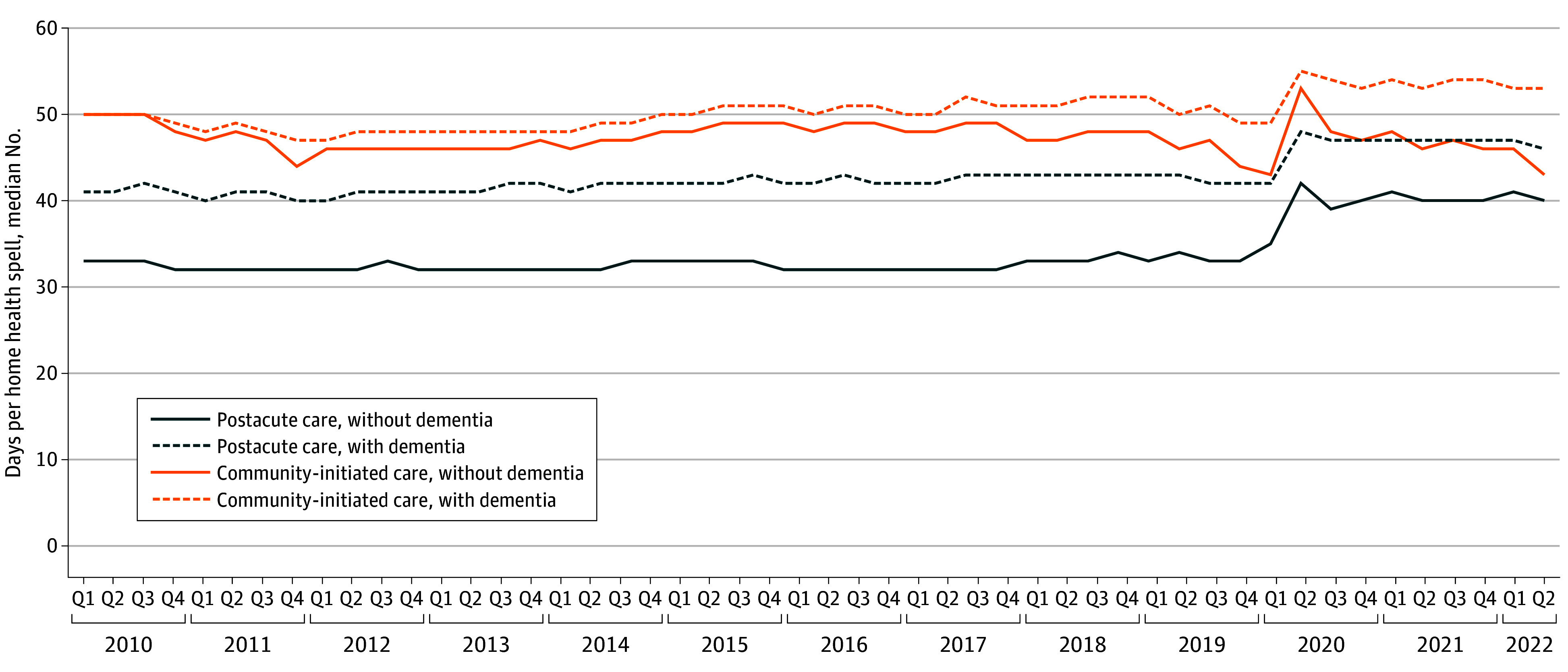
Median Length of Home Health Care Spells Among Medicare Beneficiaries With or Without a Diagnosis of Dementia Q indicates quarter.

Among those with a diagnosis of dementia, the median length of home health spells from 2010 to 2019 ranged from 40 days (IQR, 23-59 days) to 43 days (IQR, 25-59 days) for postacute care and from 47 days (IQR, 27-80 days) to 52 days (IQR, 29-89 days) for community-initiated home health care (Figure 2). For both types of home health care, spell lengths increased between 2020 and mid-2022, to a median peak of 48 days (IQR,27-59 days) for postacute care and 55 days (IQR, 33-111 days), for community-initiated home health care. The median length of home health care spells from 2010 to 2019 for individuals without a diagnosis of dementia was comparatively shorter for both postacute and community-initiated spells, ranging from 32 days (IQR, 19-56 days) to 34 days (IQR, 20-56 days) for postacute care and from 44 days (IQR, 25-76 days) to 50 days (IQR, 27-91 days) for community-initiated care. Median spells also increased in this population between 2020 and mid-2022, to a peak of 42 days (IQR, 24-58 days) for postacute care and 53 days (IQR, 27-98 days) for community-initiated care. Adjusting for sociodemographic characteristics did not qualitatively change the trends in use or medial length of home health spells (eFigure 2 and eFigure 3 in Supplement 1).

## Discussion

We found that home health care was commonly used among people with a diagnosis of dementia, particularly for community-initiated home health care. Home health care use increased in this population between 2010 and 2020 and then decreased through mid-2022. In comparison, home health care use among individuals without a diagnosis of dementia was relatively low and has been gradually decreasing.

For much of the past decade, there has been an increase in the use of home-based care as institutional care has decreased for both postacute care and long-term care.^5^ This shift toward home-based care is associated in part with pressures to constrain Medicare spending, as institutional care is relatively expensive compared with home-based care. Alternative payment models that seek to constrain spending may increase the demand for home health care services as a lower-cost alternative to institutional care.^6^ As a result, the need for support in noninstitutional settings, such as the home, may result in an increased demand for home health care. Prior research has shown that the shift toward home-based care increases the demand for help at home.^6^ In addition, receiving care at home when possible is often more consistent with patient preferences. The high rates of home health care use for individuals with Alzheimer disease and other dementias may reflect the high burden of providing care at home.

The most striking changes that we observed in home health care use occurred in 2020 with the COVID-19 pandemic, when the initiation of new home health care spells decreased precipitously. We also observed that although there was an initial rebound in home health care use in late 2020, home health care use subsequently decreased through the end of the study period in mid-2022, particularly among those with a diagnosis of dementia. In addition, the length of home health care spells increased and then remained substantially higher after the pandemic than it was before.

Although our data cannot speak to the reason for the reversal in those trends with the COVID-19 pandemic, the decreasing use of home health care is consistent with reports of widespread staffing shortages in home care agencies, which may reduce the capacity of home health agencies to meet demand for services.^8^ In addition, while patients have had a long-standing preference for receiving care at home when possible, the COVID-19 pandemic may have reduced preferences for care in the home in an effort to reduce exposure to infection.^9^ Whether these trends will continue after our study period is not known.

There was also an important change in Medicare payment for home health care starting on January 1, 2020, with the implementation of the PDGM. In addition to reducing the length of an episode of home health care from 60 to 30 days, the PDGM model adjusted payment based on clinical characteristics instead of on the amount of therapy provided.^10^ It also reduced reimbursement for community-initiated home health care.^11^ The observed changes in home health use after 2020 might also be due to these payment changes.

### Limitations

Our study includes several limitations. First, we relied on claims data for the diagnosis of dementia. In addition, the COVID-19 pandemic likely disrupted health care use such that the diagnosis of dementia was less likely to be made during that time period. Thus, toward the end of our study period, we may be underestimating who had a diagnosis of dementia. In addition, as noted, the concurrent changes to home health from the COVID-19 pandemic and the implementation of PDGM may have limited our ability to disentangle their effects. The longer-term trends after these changes are not known. However, our goal in this observational study was to describe trends in use rather than to test hypotheses about the causes of these trends. In addition, we included home health spells only for beneficiaries enrolled in traditional Medicare, as the indicator for the diagnosis of Alzheimer disease and related dementias is not available for those enrolled in Medicare Advantage, as it is based on diagnostic codes in billing data. To account for trends in home health use that may be associated with shifting sociodemographic characteristics that are associated with Medicare Advantage enrollment, we adjusted our analyses for sociodemographic characteristics and found no meaningful difference in trends. Nonetheless, Medicare Advantage enrollees are known to use home health care at lower rates and for shorter periods.^5^ Thus, the growth in Medicare Advantage enrollment over this period may have been associated with the observed trends in home health care use among those enrolled in traditional Medicare.

## Conclusions

These findings of this cross-sectional study provide new and important information about the ways in which home health care use is changing over time. They indicate a high and increasing use of help at home among people with dementia. Despite increasing use of home health care during this time period, people may receive incomplete support for their home health care needs through Medicare,^12^ which is centered on needs for skilled care, or Medicaid, which entails strict asset and income tests. Decreasing rates of home health care use since 2020 in this high-need population point to a need for ongoing monitoring of service use and outcomes for people with dementia.

## Notes

Supplement 1. **eFigure 1.** The Number of Medicare Beneﬁciaries Using Home Health, With and Without a Diagnosis of Dementia **eFigure 2.** The Number of Home Health Spells Per 1,000 Medicare Beneﬁciaries With and Without a Diagnosis of Dementia, Adjusted for Sociodemographic Characteristics **eFigure 3.** The Median Length of Home Health Spells Among Medicare Beneﬁciaries With and Without a Diagnosis of Dementia, in Days, Adjusted for Sociodemographic Characteristics

Supplement 2.
Data Sharing Statement
