# Supplementary material for: Trends in Home Health Care Among Traditional Medicare Beneficiaries With or Without Dementia
Source: JAMA Netw Open. 2025 May 16;8(5):e2510933. doi: 10.1001/jamanetworkopen.2025.10933 (PMC12084839; doi:10.1001/jamanetworkopen.2025.10933)
Supplement: Supplement 1. — eFigure 1. The Number of Medicare Beneﬁciaries Using Home Health, With and Without a Diagnosis of Dementia eFigure 2. The Number of Home Health Spells Per 1,000 Medicare Beneﬁciaries With and Without a Diagnosis of Dementia, Adjusted for Sociodemographic Characteristics eFigure 3. The Median Length of Home Health Spells Among Medicare Beneﬁciaries With and Without a Diagnosis of Dementia, in Days, Adjusted for Sociodemographic Characteristics [file jamanetwopen-e2510933-s001.pdf]

Supplementary Online Content

Werner RM, Kim S, Konetzka RT. Trends in home health care among traditional Medicare beneficiaries with or without dementia. *JAMA Netw Open.* 2025;8(5):e2510933. doi:10.1001/jamanetworkopen.2025.10933

eFigure 1. The Number of Medicare Beneficiaries Using Home Health, With and Without a Diagnosis of Dementia
eFigure 2. The Number of Home Health Spells Per 1,000 Medicare Beneficiaries With and Without a Diagnosis of Dementia, Adjusted for Sociodemographic Characteristics
eFigure 3. The Median Length of Home Health Spells Among Medicare Beneficiaries With and Without a Diagnosis of Dementia, in Days, Adjusted for Sociodemographic Characteristics

This supplementary material has been provided by the authors to give readers additional information about their work.

© 2025 Werner RM et al. *JAMA Network Open.*

eFigure 1. The Number of Medicare Beneficiaries Using Home Health, With and Without a Diagnosis of Dementia

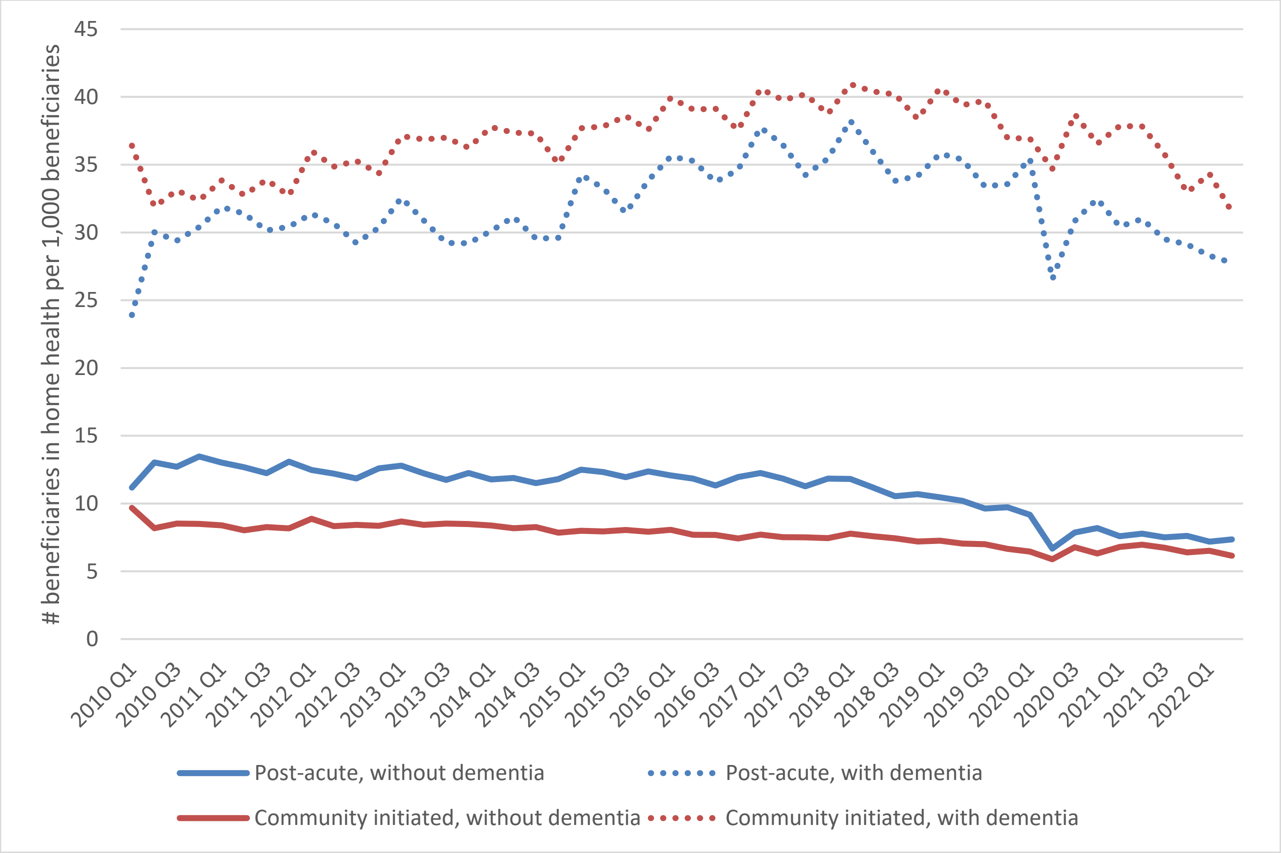

© 2025 Werner RM et al. *JAMA Network Open.*

eFigure 2. The Number of Home Health Spells Per 1,000 Medicare Beneficiaries With and Without a Diagnosis of Dementia, Adjusted for Sociodemographic Characteristics

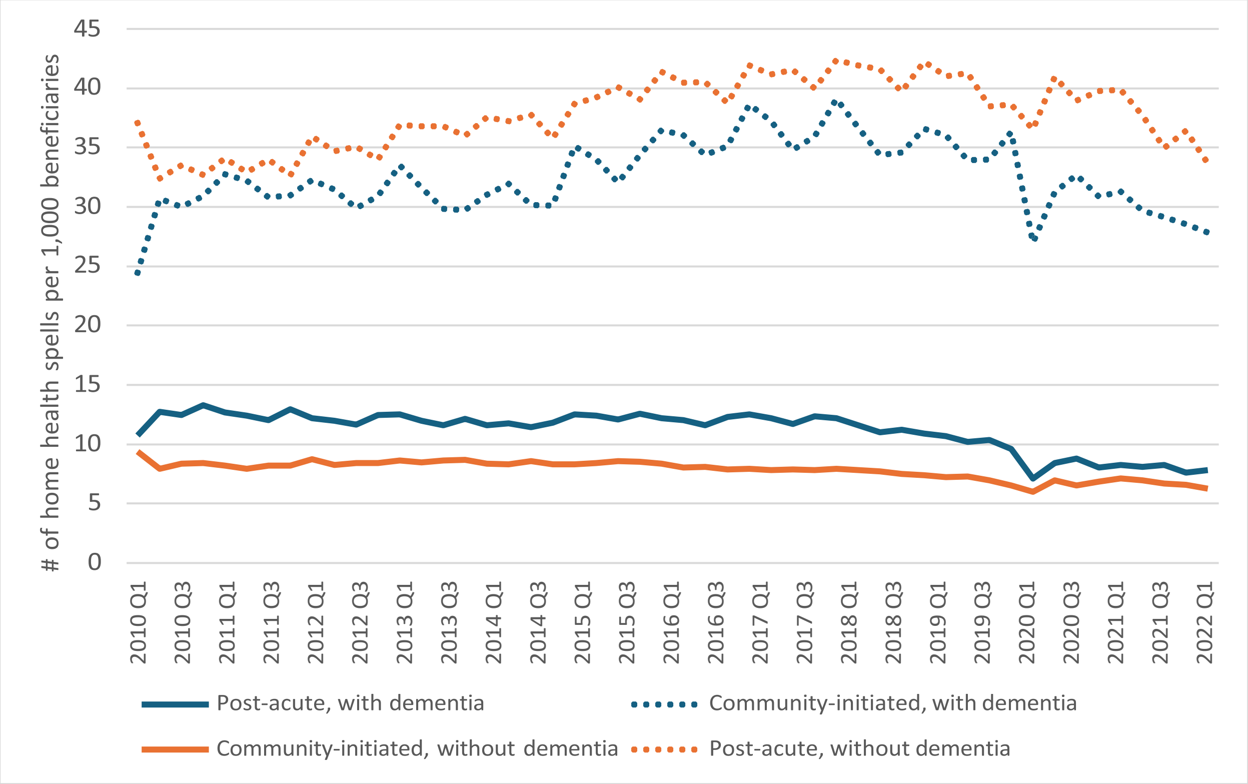

© 2025 Werner RM et al. *JAMA Network Open.*

eFigure 3. The Median Length of Home Health Spells Among Medicare Beneficiaries With and Without a Diagnosis of Dementia, in Days, Adjusted for Sociodemographic Characteristics

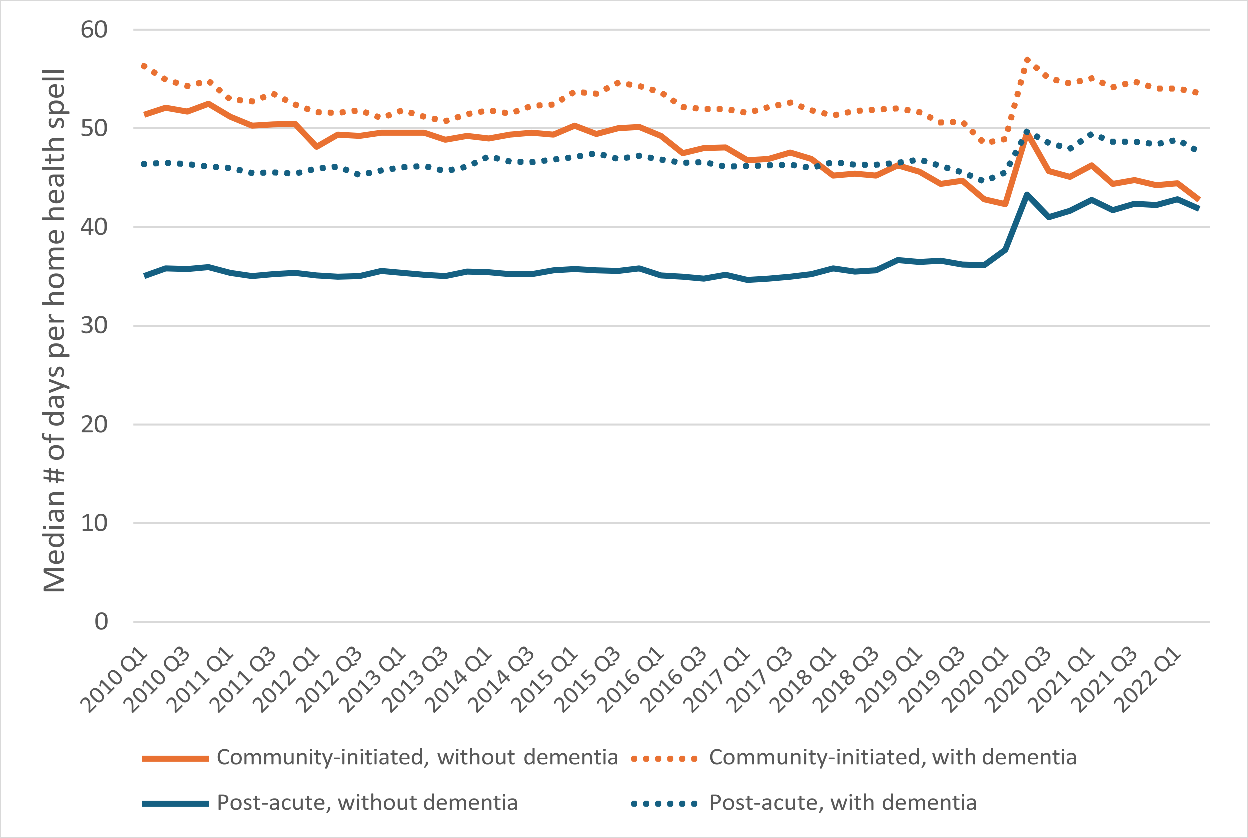

© 2025 Werner RM et al. *JAMA Network Open.*
