# Supplementary material for: Trends in Home Health Care Among Traditional Medicare Beneficiaries With or Without Dementia
Source: JAMA Netw Open. 2025 May 16;8(5):e2510933. doi: 10.1001/jamanetworkopen.2025.10933 (PMC12084839; doi:10.1001/jamanetworkopen.2025.10933)
Supplement: Supplement 2. — Data Sharing Statement [file jamanetwopen-e2510933-s002.pdf]

Data Sharing Statement

Werner. Trends in Home Health Care Among Traditional Medicare Beneficiaries With or Without Dementia. *JAMA Netw Open*. Published May 16, 2025.
doi:10.1001/jamanetworkopen.2025.10933

Data
Data available: No

Additional Information: RIF Medicare files are not able to be shared. Code to create the data files is available at https://github.com/rwerner-upenn/Recent-trends-in-home-health-care-among-traditional-Medicare-beneficiaries-with-and-without-dementia
Explanation for why data not available: RIF Medicare files are not able to be shared. Code to create the data files will be shared
